# Characteristics and clinical outcomes of patients with acute gastrointestinal bleeding related to anticoagulant or antiplatelet therapy: a retrospective study

**DOI:** 10.3325/cmj.2021.62.488

**Published:** 2021-10

**Authors:** Dorotea Božić, Jonatan Vuković, Ivona Mustapić, Pavle Vrebalov Cindro, Joško Božić, Goran Kardum, Željko Puljiz, Ivana Tadin Hadjina, Ante Tonkić

**Affiliations:** 1 Department of Gastroenterology and Hepatology, University Hospital Split, Split, Croatia; 2 University of Split, School of Medicine, Split, Croatia; 3 Department of Cardiology, University Hospital Split, Split, Croatia; 4 Department of Pathophysiology, University of Split, School of Medicine, Split, Croatia; 5 Faculty of Humanities and Social Sciences, University of Split, Split, Croatia

## Abstract

**Aim:**

To investigate the demographic characteristics, endoscopic and laboratory findings, comorbidities and mortality rate of patients with gastrointestinal bleeding related to anticoagulant or antiplatelet therapy.

**Methods:**

We reviewed the records of patients admitted for gastrointestinal bleeding to the Intensive Care Unit of the Department of Gastroenterology, University Hospital Split, between 2015 and 2019. The characteristics and clinical outcomes of patients taking anticoagulant/antiplatelet therapy were analyzed.

**Results:**

The study enrolled 1367 patients, 434 (31.7%) of whom received anticoagulant/antiplatelet therapy (mean age 74.9 ± 10.7 years; 64.3% men). The most frequently prescribed drug was acetylsalicylic acid (56.7%), the most common bleeding site was the stomach (41.3%), and the most prevalent cause of bleeding was ulcer (61.6%). Patients taking anticoagulant/antiplatelet therapy who died had significantly higher creatinine (*P* = 0.011) and lower albumin (*P* = 0.015). In the multivariate analysis, the factors that negatively affected survival were older age, higher creatinine, and lower albumin. Patients taking anticoagulant/antiplatelet therapy had slightly lower in-hospital mortality (8.3%) compared with other patients (10.3%).

**Conclusion:**

Although anticoagulant/antiplatelet therapy increases the risk of gastrointestinal bleeding, it does not directly affect the outcome, which is mainly determined by age and comorbidities.

An increase in the number of patients with cardiovascular diseases is accompanied by an increased prescription of anticoagulant (AC) and antiplatelet (APT) therapy. This type of therapy is a risk factor for gastrointestinal bleeding (GIB), mainly from the upper gastrointestinal tract. Upper GIB affects up to 10% of patients, with an annual risk of 1.5%-4.5% (1). APT therapy is a basic treatment in the primary and secondary prophylaxis of adverse cardiovascular events, such as myocardial infarction and ischemic stroke. Patients with atherosclerotic disease mainly receive acetylsalicylic acid (ASA), a mainstay of prophylactic treatment, followed by adenosine diphosphate P2Y_12_ receptor blockers, which include clopidogrel and ticagrelor (2).

The indications for long-term anticoagulant therapy are prosthetic heart valves, atrial fibrillation (AF), deep venous thrombosis, hypercoagulable diseases, and vascular diseases, with major gastrointestinal bleeding events reported in up to 20% patients (3). Novel oral anticoagulants (NOACs), which include direct thrombin inhibitor (dabigatran) and direct factor Xa inhibitors (rivaroxaban, apixaban), have been used for the prevention of embolic stroke in non-valvular atrial fibrillation and the prevention and treatment of venous thromboembolism (4).

The mortality rate of GIB during AC and/or APT therapy has been underexplored. Therefore, the primary aim of our study was to compare the outcomes between patients taking AC and/or APT therapy and patients without AC and/or APT therapy, and to investigate the effects of demographic characteristics, endoscopic findings, laboratory values, and comorbidities on the survival in the AC/APT group. The secondary aim was to compare the bleeding localization, bleeding source, and survival between various drug classes.

## PATIENTS AND METHODS

### Patients

We retrospectively reviewed the records of patients treated with AC or APT therapy admitted for GIB to the Intensive Care Unit (ICU) of the Department of Gastroenterology and Hepatology, University Hospital Center Split, between January 1, 2015 and December 31, 2018. We identified 1367 patients with upper and lower GIB, 434 of whom were treated with AC (warfarin and NOACs including apixaban, rivaroxaban, and dabigatran) and/or APT therapy (ASA, clopidogrel, ticagrelor) at admission.

### Methods

Data were collected for patients admitted under the diagnosis of GIB (ICD-10 K92.2), melena (ICD-10 K92.1), and hematemesis (ICD-10 K92.0). The indications for ICU admission were hematemesis, melena, and moderate/severe hematochezia. Patients admitted for severe anemia, suspected GIB that was excluded upon admission, and iatrogenic bleeding (eg, post-polypectomy bleeding) were not included.

We gathered data on age, sex, type of AC/APT therapy, bleeding localization (esophagus, stomach, duodenum, large intestine), and bleeding source (varices, ulcer, polyp, angiodysplasias, diverticles, neoplasm, hemorrhoids). The analyzed laboratory findings were red blood cell count (RBC), platelet count (PLT), hemoglobin, international normalized ratio (INR), activated partial thromboplastin time (aPTT), albumin, creatinine, aspartate aminotransferase (AST), and alanine aminotransferase (ALT). The analyzed comorbidities were ischemic heart disease, heart failure, diabetes mellitus (DM), arterial hypertension (AH), and atrial fibrillation (AF). In-hospital outcome was defined as a death or survival.

All procedures conformed to the ethical standards of the institutional and national research committee and the 1964 Helsinki declaration and its later amendments or comparable ethical standards. The ethical approval and informed consent were waived by the Ethics Committee of Split University Hospital in view of the retrospective study design and since all the procedures were part of the routine patient care.

### Statistical analysis

The normality of distribution was assessed with the Kolmogorov-Smirnov test. Continuous variables are presented as mean ± standard deviation or median and range, while categorical variables are presented as counts and percentages (%). The *t* test for independent samples and the Mann-Whitney test were used to compare the continuous variables, while the χ^2^ test and Fisher exact test were used for the categorical variables. Multivariate logistic regression adjusted for age and sex was used to determine independent predictors of survival. Multivariate-adjusted odds ratio (OR) and 95% confidence intervals (CI) are reported. The significance level was set at *P* < 0.05. The statistical analysis was performed with MedCalc software, version 17.9.4, (MedCalc Software, Ostend, Belgium).

## RESULTS

### Patient characteristics

The study enrolled 1367 patients, 434 (31.7%) in the AC/APT group (279 or 64.3% men). Upon admission, 192 patients were taking AC (44.2%) and 251 were taking APT (57.8%). Only nine patients (2%) were taking both therapies.

The mean age in the AC/APT group was 74.9 ± 10.7 years. The unexposed group was significantly younger (67.7 ± 15.7) (*P* < 0.001), but did not significantly differ from the AC/APT group in sex distribution (67.6% men) (*P* = 0.402). The most frequently prescribed drug was ASA (246 patients or 56.7%), followed by warfarin (152 patients or 35%). Forty patients (9.2%) received NOACs. The fewest patients (23 or 5.3%) were taking clopidogrel or ticagrelol, with 18 of these patients being on dual antiaggregation therapy.

### Bleeding localization and causes

Out of 434 patients, 397 had an endoscopically determined bleeding site. The most frequent bleeding site was the stomach (41.3%), followed by the large intestine (25.7%), duodenum (24.9%), and esophagus (8.1%) (Table 1).

**Table 1:** Mortality in the anticoagulant or antiplatelet therapy group regarding the bleeding site and cause; data are counts and percentages

	N (%)	Fatal outcome	P*
**Site**			
esophagus	32 (8.1)	0 (0)	0.139
stomach	164 (41.3)	15 (9.1)	
duodenum	99 (24.9)	7 (7.1)	
large intestine	102 (25.7)	5 (4.9)	
not defined	37 (8.5)		
total	434 (100)	27 (6.8)	
**Cause**			
varices	21 (5.7)	0 (0)	0.124
ulcer	228 (61.6)	21 (9.2)	
polyp	23 (6.2)	1 (4.4)	
angiodysplasia	8 (2.2)	0 (0)	
diverticulum	29 (7.8)	0 (0)	
neoplasm	30 (8.1)	0 (0)	
hemorrhoids	31 (8.4)	2 (6.5)	
not found	64 (14.75)		
total	434 (100)	24 (6.5)	

*χ^2^ test.

The bleeding cause was successfully identified in 370 patients. The most common was ulcer (61.6%). None of the other causes (neoplasms, hemorrhoids, diverticles, polyps, and angiodysplasias) individually caused more than 9% of all bleedings (Table 1).

The most frequent cause of upper GIB were stomach ulcers (n = 130) and duodenal ulcers (n = 92). The most common causes of lower GIB were hemorrhoids (31 patient) and diverticles (29 patients).

The most common bleeding sites in the APT group were the stomach (36.3%) and duodenum (33.3%), and in the AC group, the stomach (49.7%) and large intestine (30.2%), with a similar distribution for both warfarin and NOACs. In the univariate analysis, the bleeding site and pathological substrates did not significantly affect the survival.

### Comorbidities

Most patients had AH (62%), followed by ischemic heart disease (45%), heart failure (34%), AF (32%), and DM (23%). In the univariate analysis, surviving and non-surviving patients did not differ in the distribution of comorbidities. Interestingly, diabetic (9%) and non-diabetic (8.06%) patients did not differ according to the number of fatal outcomes (*P* = 0.759). Patients with heart failure more frequently died (11.41%) compared with patients without heart failure (6.64%, *P* = 0.085); similar to patients with and without AF (11.43% vs 6.8%, *P* = 0.103). Surprisingly, patients with AH less frequently died (6.69%) when compared with the group without AH (10.91%), but the difference (*P* = 0.127) was not significant in the univariate analysis.

### Laboratory parameters

RBC, PLT, hemoglobin, INR, aPTT, and gamma glutamyl transferase (GGT) values did not significantly differ regarding the outcome. Non-survivors compared with survivors had higher activated partial thromboplastin time and INR, but the difference was not significant. Non-survivors had significantly higher AST, ALT and creatinine values, as well as lower albumin values (Table 2).

**Table 2:** Demographic characteristics and laboratory parameters of patients treated with anticoagulant or antiplatelet therapy. Data are means ± standard deviations or medians (interquartile ranges)

Characteristic	Surviving patients	Non-surviving patients	*P*
Mean age (years)	74.38 ± 10.55	81.64 ± 10.74	<0.005^‡^
Female sex*	140 (90.3)	15 (9.7)	0.375^†^
Male sex*	258 (92.5)	21 (7.5)	
Hemoglobin (g/L)	96.28 ± 27.44	94.86 ± 32.47	0.77^‡^
Red blood cell count ( × 10^12^/L)	3.29 ± 0.9	3.27 ± 1.07	0.885^‡^
Platelet count ( × 10^9^/L)	215.5 (167.0-288.0)	228.5 (157.0-303.0)	0.949^§^
International normalized ratio	1.12 (0.98-2.4)	1.23 (1.05-3.1)	0.067^§^
Activated partial thromboplastin time (s)^I^	26.0 (22.2-35.1)	27.0 (22.9-44.5)	0.350^§^
Albumin (g/L)	30.36 ± 7.43	25.15 ± 5.2	0.015^‡^
Creatinine (μmol/L)	85.0 (68.0-121.0)	136.0 (77.5-192.0)	0.003^§^
Aspartate transaminase (IU/L)	18.0 (14.0-25.0)	28.0 (18.2-65.0)	0.001^§^
Alanine transaminase (IU/L)	16.0 (11.0-25.0)	20.0 (14.2-43.7)	0.018^§^
Gamma glutamyl transferase (IU/L)	24.0 (15.0-57.0)	33.5 (18.0-73.0)	0.149^§^

*Counts and percentages.

†χ^2^ test.

‡t-test.

§Mann-Whitney U test.

### Outcomes

**Figure 1:**
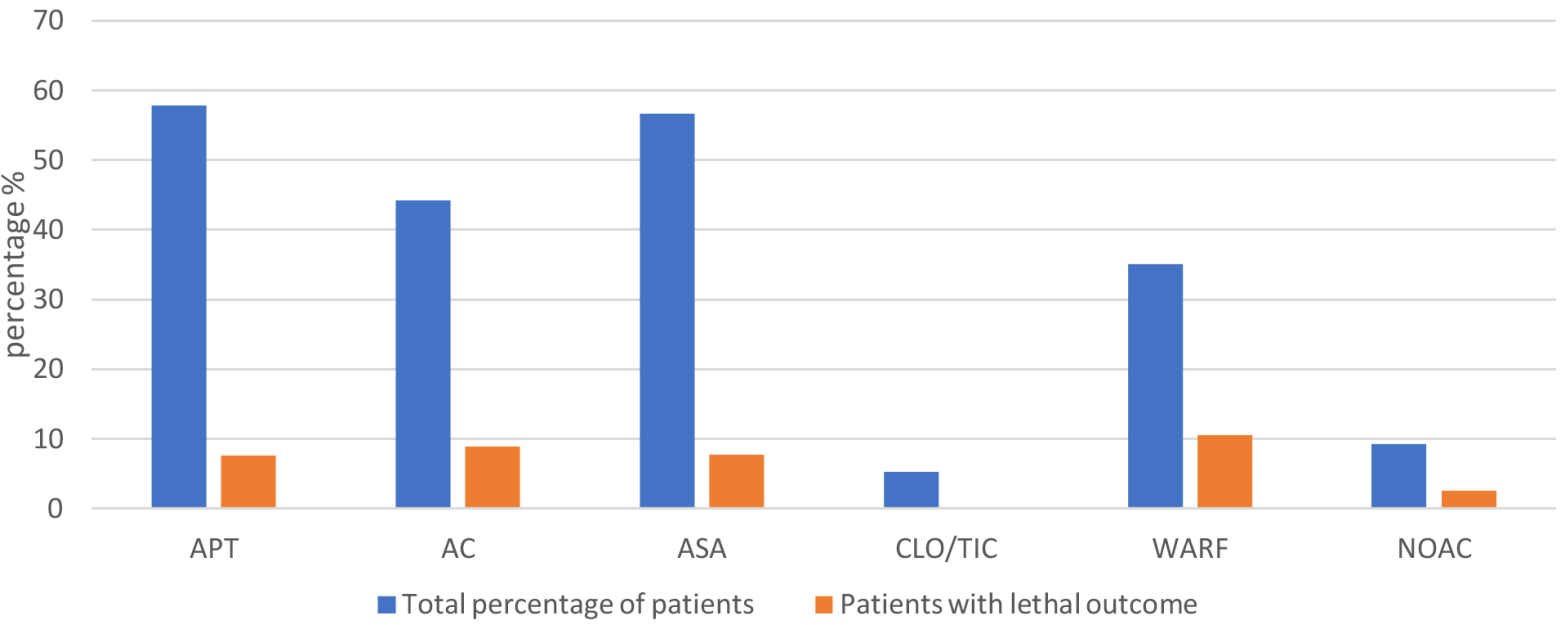
The mortality rates according to the type of treatment in the anticoagulant or antiplatelet therapy group. APT – antiplatelet therapy, AC – anticoagulant therapy, ASA – acetylsalicylic acid, CLO/TIC – clopidogrel/ticagrelol, WARF – warfarin, NOAC – new oral anticoagulants.

Overall in-hospital mortality was 9.7%: 8.9% for men and 11.2% for women. Among 434 patients in the AC/APT group, in-hospital mortality was 8.3%, which was lower than in the non-exposed group (10.3%), but the difference was not significant (*P* = 0.228). In the AC/APT group, there was no significant difference in survival between the sexes (Table 2). In-hospital mortality in patients treated with APTs was 7.6%, and 8.9% in patients treated with ACs. Patients treated with NOACs had the lowest mortality rate (2.5%) compared with other drugs, but the difference was not significant (*P* = 0.232) (Figure 1).

In the multivariate analysis, survival was significantly affected by age (*P* = 0.009, odds ratio [OR] 1.082, 95% confidence interval [CI] 1.019-1.149), creatinine levels (*P* = 0.045, OR 1.005, 95% CI 1.0001-1.010), and albumin levels (*P* = 0.025, OR 0.895; 95% CI 0.813- 0.986).

## DISCUSSION

In our study, AC/APT therapy did not affect the outcomes of patients admitted to the ICU for a GIB event.

APT therapy is mainly used for treatment of cardiovascular diseases and ischemic cerebrovascular insult. ASA treatment is a well-known risk factor for GIB (5,6). Although clopidogrel was shown to have a significantly better safety profile regarding bleeding events due to weak gastric mucosal inflammation, in combination with ASA, it increased the risk of upper GIB more than when ASA was given alone (6-8). Still, adding proton pump inhibitors to ASA better prevented against upper GIB recurrences than switching to clopidogrel (9).

AC therapy is mainly used for treatment of AF, thromboembolic events, hypercoagulable diseases, and in patients with prosthetic heart valves. Widely used vitamin K antagonists are slowly being replaced by NOACs, which do not require routine hemostasis testing. The risk of thromboembolic and bleeding events is routinely assessed with CHA2DS-VASc and HASBLED scores, respectively. The multicenter prospective PREFER study (10) found that thromboembolic and major bleeding events in patients on AC therapy were independently associated with abnormal liver function, prior stroke or transient ischemic attack, labile INR, concomitant therapy with APT or non-steroidal antirheumatics (NSAR), heart failure, and older age. This indicates that modifiable risk factors should be targeted in order to reduce bleeding events in anticoagulated patients (10).

A meta-analysis by Miller et al (11) showed no difference between NOACs and vitamin K antagonists in the risk of major bleeding. On the other hand, Adisaksopha et al (12) found NOACs to be associated with a lower risk of major bleeding, fatal bleeding, intracranial bleeding, clinically relevant non-major bleeding, and total bleeding. Still, they found no difference between NOACs and vitamin K antagonists in the risk of GIB, an observation that was later confirmed by other meta-analyses (11,13,14). Caldeira et al (13) found a similar bleeding risk between NOACs and ASA in patients with AF, and no significant difference between NOACs and low-molecular-weight heparin (LMWH) in patients undergoing a major orthopedic surgery. It is important to notice that Miller et al (11) found dabigatran and rivaroxaban to be associated with an increased odds of a major GIB. In the case of dabigatran, tartaric acid coating has been proposed to directly negatively affect the intestinal lumen, therefore enhancing the bleeding risk (11). A meta-analysis in patients aged ≥75 years found GIB to be significantly increased with dabigatran 150 mg and 110 mg, in comparison with vitamin K antagonists (15). Burr et al proved factor Xa inhibitors to carry a reduced risk of GIB compared with warfarin and dabigatran (14).

Approximately 26% of patients hospitalized for upper GIB were treated with AC or APT therapy, and this percentage reached 50.7%-59.3% when patients treated with NSAR were taken into account (1,6,16,17). Oakland et al (18) demonstrated a high exposure to AC or APT therapy (36.3%) in patients admitted for lower GIB. In our study, a considerable percentage (31.7%) of patients received APT or AC therapy at the time of a bleeding event. These patients also had a high mean age (74.9 ± 10.7 years). Other studies also observed a high mean age (67.7-77.1 years) in the APT or AC group (1,3,6,19,20).

Similar to the present study, other authors found that the most prevalent bleeding lesion was peptic ulcer, and that the most common bleeding-related agent was ASA (1,3,16,20,21). In our study, the most frequent bleeding site was gastric ulcer, confirming the decrease in duodenal ulcer bleedings over the years (22).

In agreement with the study by Pannach et al (23), we found upper GIB to be most frequently caused by APTs. In addition, AC therapy caused more upper (69.8%) than lower (30.2%) GIB events. Pannach et al also found vitamin K antagonists to more often cause upper GIB, although in a lower percentage than in our study (53% vs 70.5%). Studies reported NOACs-associated upper GIB to be more severe than lower GIB, which in most cases is caused by hemorrhoids (21,23). Most bleeding incidents occurred in the first year after NOACs initiation (21).

The number of patients with cardiovascular, pulmonary, or renal comorbid diseases significantly increased over the years (22). In our cohort, the most common comorbidities were AH (62%), ischemic heart disease (45%), heart failure, (34%), AF (32%), and DM (23%). Certain upper GIB studies in patients receiving APT/AC treatment also reported a high prevalence of AH and cardiovascular diseases, but also of DM (1,16,20). In the study by Jorgensen et al, DM and AF were identified as risk factors for a fatal outcome in univariate analysis, but in multivariate analysis none of the variables remained significant (1). In a study by Sampaio et al, the risk of 30-day mortality was five times higher in patients with one or more comorbidities, while metastatic disease increased the risk 12-fold (20).

In our study, high creatinine and low albumin were the only laboratory parameters identified in the multivariate analysis to affect the survival. Low albumin level might be caused by liver failure or malnutrition/malapsorption, and is often observed during inflammatory response. Lower albumin concentration is also observed in the elderly and in patients with heart failure due to intestinal edema that disables an adequate albumin absorption. Therefore, low albumin usually indicates a comorbid condition. Furthermore, hypoalbuminemia increases free drug levels, which is noteworthy since the majority of anticoagulants are protein bound.

High creatinine value indicates renal failure. Prerenal failure commonly occurs due to dehydration, hypovolemia in a severe bleeding event, low preload caused by heart failure, or renal hypoperfusion during the septic course. High creatinine therefore signals chronic kidney disease and/or other significant comorbidities, and leads to impaired drug excretion. This could explain the significant effects of albumin and creatinine values on the mortality in our study group.

We observed an overall in-hospital mortality of 9.7%. Interestingly, the mortality was slightly lower in the AC/APT group (8.3%) than in nonexposed group (10.3%). Similar results were observed in other studies (3,20). We found no significant difference in overall mortality according to sex or type of therapy. Studies report varying mortality rates in patients with upper GIB receiving AC/APT (3.5%-8.8%) (1,3,6,20). A study including patients with both upper and lower GIB documented a mortality rate ranging from 1.6% in patients treated with NOACs to 11.9% in patients treated with APTs (23). The mortality rate between 3% and 10% seems to be unchanged despite the advancement in endoscopic procedures. A possible reason is a higher mean age of patients combined with a wide spectrum of comorbidities, since the majority of deaths are related to non-bleeding events.

Study limitations include the retrospective study design, a single-center setting, a lack of long-term follow-up, and a low percentage of patients receiving certain drug types (eg, clopidogrel, ticagrelol, NOACs). Since the capsule endoscopy was usually performed following the hospitalization, small bowel bleedings were not detected.

In conclusion, this study demonstrated a high exposure rate to APT and AC therapy (31.7%) in a large cohort of patients admitted for both upper and lower GIB. Older age and the presence of certain comorbidities (renal failure, hypoalbuminemia) were the main predictors of death, regardless of the drug type or bleeding localization or cause. Patients with these risk factors are recommended to undergo bleeding control with advanced endoscopic methods and receive treatment for comorbidities. Slightly, although not significantly, lower rates of in-hospital mortality in the ATP/AC group indicate that although AC and APT increase the risk of GIB, they do not directly affect the outcome. Still, we must not disregard the fact one third of bleeding events and possibly their undesirable outcomes were related to AC or APT therapy. Therefore, it is advocated to carefully weigh the benefits and side effects of this type of treatment, taking into consideration the patients' age and comorbidities. Further studies focusing on patients with GIB taking NOACs, and long-term prospective studies are warranted.
